# Glycerol-3-Phosphate Acyltranferase-2 Behaves as a Cancer Testis Gene and Promotes Growth and Tumorigenicity of the Breast Cancer MDA-MB-231 Cell Line

**DOI:** 10.1371/journal.pone.0100896

**Published:** 2014-06-26

**Authors:** Magali Pellon-Maison, Mauro A. Montanaro, Ezequiel Lacunza, Maria B. Garcia-Fabiani, Mercedes C. Soler-Gerino, Elizabeth R. Cattaneo, Ivana Y. Quiroga, Martin C. Abba, Rosalind A. Coleman, Maria R. Gonzalez-Baro

**Affiliations:** 1 Instituto de Investigaciones Bioquímicas de La Plata, Consejo Nacional de Investigaciones Científicas y Técnicas, Facultad de Ciencias Médicas, Universidad Nacional de La Plata. La Plata, Argentina; 2 Centro de Investigaciones Inmunológicas Básicas y Aplicadas, Facultad de Ciencias Médicas, Universidad Nacional de La Plata, La Plata, Argentina; 3 Department of Nutrition, University of North Carolina, Chapel Hill, North Carolina, United States of America; Wayne State University School of Medicine, United States of America

## Abstract

The *de novo* synthesis of glycerolipids in mammalian cells begins with the acylation of glycerol-3-phosphate, catalyzed by glycerol-3-phosphate acyltransferase (GPAT). GPAT2 is a mitochondrial isoform primarily expressed in testis under physiological conditions. Because it is aberrantly expressed in multiple myeloma, it has been proposed as a novel cancer testis gene. Using a bioinformatics approach, we found that GPAT2 is highly expressed in melanoma, lung, prostate and breast cancer, and we validated GPAT2 expression at the protein level in breast cancer by immunohistochemistry. In this case GPAT2 expression correlated with a higher histological grade. 5-Aza-2′ deoxycytidine treatment of human cells lines induced GPAT2 expression suggesting epigenetic regulation of gene expression. In order to evaluate the contribution of GPAT2 to the tumor phenotype, we silenced its expression in MDA-MB-231 cells. GPAT2 knockdown diminished cell proliferation, anchorage independent growth, migration and tumorigenicity, and increased staurosporine-induced apoptosis. In contrast, GPAT2 over-expression increased cell proliferation rate and resistance to staurosporine-induced apoptosis. To understand the functional role of GPAT2, we performed a co-expression analysis in mouse and human testis and found a significant association with semantic terms involved in cell cycle, DNA integrity maintenance, piRNA biogenesis and epigenetic regulation. Overall, these results indicate the GPAT2 would be directly associated with the control of cell proliferation. In conclusion, we confirm GPAT2 as a cancer testis gene and that its expression contributes to the tumor phenotype of MDA-MB-231 cells.

## Introduction

The *de novo* synthesis of glycerolipids in mammalian cells begins with the acylation of glycerol-3-phosphate, catalyzed by glycerol-3-phosphate acyltransferase (GPAT) [1]. As occurs in many other lipid metabolic reactions, several isoforms catalyze this step. At least four different genes encode for GPAT isoforms 1–4, which differ in tissue expression pattern, subcellular localization, fatty acyl-CoA substrate preference, and sensitivity to N-ethylmaleimide. GPAT1 and GPAT2 are mitochondrial isoforms, whereas GPAT3 and GPAT4 are localized in the endoplasmic reticulum [2]. While GPAT1, GPAT3 and GPAT4 are expressed in lipogenic tissues and their activities are associated with triacylglycerol and phospholipid synthesis, the expression pattern of GPAT2 is more prominent in testis [3]. GPAT2, which is expressed in the germ line cells in mouse and rat testis, is highly selective for arachidonoyl-CoA as a substrate [4]. The *Gpat2* gene is transcribed only in primary spermatocytes and the level of both mRNA and protein decreases in subsequent steps of the spermatogenic cycle. The function of GPAT2 in male reproduction remains unknown, but a recent publication showed that GPAT2 is essential for the biogenesis of piRNA which maintains genome integrity in germ line cells [5].

Based on a study of multiple myeloma, GPAT2 was proposed to be a novel “cancer-testis” gene (CT gene) candidate [6]. CT genes encode proteins whose expression is restricted to male germ cells and to several tumors of different histological origins, but CT gene products are absent or expressed at a low level in normal somatic cells [7]. Their expression is usually regulated by epigenetic mechanisms, and they are immunogenic. Due to their immunogenic properties, growing lists of CT antigens are being considered as targets for cancer vaccines [8]. However, little is known about the function of CT gene products in either spermatogenic or malignant cells.

The aim of this study was to determine whether GPAT2 behaves as a CT gene and to evaluate the phenotypic consequence of GPAT2 expression in cancer cells. We chose the MDA-MB-231 cell line derived from human breast carcinoma because these cells express high levels of GPAT2. GPAT2 gene knockdown in this cancer cell model showed that GPAT2 can promote cell tumorigenicity, proliferation and survival.

## Experimental Procedures

### Ethics Statement

The studies performed with nude mice were approved by the Directive Board of the INIBIOLP and were carried out in accordance with the AVMA Animal Welfare Policies (http://www.avma.org/issues/animal_welfare/policies.asp) and AVMA Guidelines on Euthanasia (http://www.avma.org/issues/animal_welfare/euthanasia.pdf). (INIBIOLP's Animal Welfare Assurance No. A5647–01).

#### Cell lines

Human breast adenocarcinoma MDA-MB-231 and colorectal adenocarcinoma HCT116 cells were purchased from the American Type Culture Collection [9] (Manassas, VA, USA). Stable cell lines expressing a small-hairpin RNA targeting GPAT2 mRNA (shRNA-GPAT2) and a non-silencing scrambled RNA (shRNA-scr) were obtained in our laboratory on the commercial MDA-MB-231 and HCT116 cell lines using routine techniques as described below.

### Bioinformatics analysis

1. Transcriptional profile of GPAT2 in human normal tissues and cancer cell lines: to evaluate GPAT2 mRNA expression in human normal tissues, we analyzed a genome wide gene expression profile of 677 samples (InSilicoDB, GSE7307). This data set comprises normal and diseased tissues and cell lines. Therefore, samples of diseased tissues and cell lines were excluded from the analysis. In addition, to obtain a more general representation of the different tissues, we combined those samples corresponding to different locations of the encephalon (thalamus, midbrain, caudate, etc.) under the single category designated as “brain.” We also consolidated samples with synonymous names, such as breast and mammary gland and omitted tissues represented by just a single sample. A filtered dataset of 36 normal human tissues was used.

In the search for an *in vitro* model in which to study the role of GPAT2 in cancerous cells, we assessed the mRNA expression of GPAT2 in a dataset of 174 samples from 59 cell lines from 9 different cancer tissues. (InSIlicoDB, GSE32474).

2. Transcriptional profile of GPAT2 across human tumor samples: to perform a comparative analysis of GPAT2 mRNA expression in different human cancers, we combined ten independent oligo-microarray studies available in a public database. To generate a homogeneous dataset, the frozen robust multiarray analysis (fRMA) preprocessed expression matrixes of the studies GSE37642 (Acute myeloid leukemia, AML), GSE7553 (primary melanoma, metastatic melanoma, squamous and basal cell carcinomas), GSE31684 (bladder carcinoma), GSE9843 (hepatocellular carcinoma), GSE18842 (lung cancer), GSE14333 (colorectal cancer), GSE21653 (breast cancer), GSE20685 (breast cancer), GSE17591 (prostate cancer), and GSE39671 (chronic lymphocytic leukemia, CLL) were downloaded from the InSilico database (http://insilico.ulb.ac.be/) [10]. These gene expression profiles were all developed with the Affymetrix HG U133 Plus2 platform (GPL570). Only tumor samples were considered and control and/or normal samples present in some datasets were excluded. The frozen Robust Multiarray Analysis (fRMA) pre-processing algorithm allows analysis of independent oligo-microarray studies/batches, and then combines the data for further statistical analysis [11]. Our final compiled gene expression data were 1693 cancer samples. Expression values for GPAT2 were ordered increasingly, and divided into 3-quantile distribution, identifying three groups in terms of expression levels: low, moderate and high. Quantiles are points taken at regular intervals from the cumulative distribution function of a random variable. The 3-quantiles are called terciles. In this way, one-third of all the ranked observations are smaller than the first tercile (this category was termed low), one-third lie between the first and second tercile (this category was termed moderate), and one-third are larger than the second tercile (this category was termed high).

3. GPAT2 co-expression analysis in testis: to further analyze functional pathways associated with GPAT2, we employed the ‘Guilt by association’ principle, which states that gene co-expression might indicate shared regulatory mechanisms and roles in related biological processes [12]. Because GPAT2 resides principally in testis tissue, co-expressed genes in mouse and human testis were obtained by using the web-based bioinformatics tool Multiexperiment Matrix (MEM) http://biit.cs.ut.ee/mem/ [13]. We selected the 300-best positively correlated genes (p<0.0001) and performed the functional enrichment analysis using the DAVID [14] and REVIGO [15] tools.

### Cell lines and culture conditions

The human MDA-MB-231, HeLa, HEK293, MCF7 and HCT116 cell lines, derived from mammary adenocarcinoma, cervix adenocarcinoma, embryonic kidney and colorectal adenocarcinoma, respectively, and the normal monkey kidney Vero cell line were purchased from ATCC and maintained in DMEM supplemented with 10% FBS, 100 U/ml penicillin, 100 mg/ml streptomycin and 2 mM glutamine. Cells were grown at 37°C in a 5% CO2 atmosphere with 98% relative humidity.

### GPAT2 silencing

For human GPAT2 silencing, MDA-MB-231 and HCT116 cells were transfected using Lipofectamine 2000 Reagent (Life Technologies) with HuSH-29 plasmid (OriGene) coding for shRNA against human GPAT2 mRNA, and selected for puromycin resistance to generate the respective shRNA-GPAT2 cell line. A non-effective scrambled sequence shRNA plasmid was used to create a negative control for each cell line (shRNA-Scr). Both plasmids also contain a sequence coding for green fluorescent protein driven by a CMV promoter. GPAT2 knock down was assessed by QPCR.

### Quantitative Real-time PCR

Total RNA was isolated from cell lines using TRIZOL (Life Technologies) following the manufacturer's instructions, and 1 µg RNA was used for cDNA synthesis employing High Capacity Reverse Transcription Kit (Applied Biosystems). A 1/10 cDNA dilution was used for the QPCR reaction with IQ Sybr Green Super Mix (Bio-Rad). Primers were designed to amplify a fragment between exon 15 (forward primer: ATCCTACTGCTGCTGCACCT) and exon 17 (reverse primer ACAGCAGCTTTGCACTCAGA) of human GPAT2. The thermal profile was 50°C for 10 min, 95°C for 5 min, followed by 40 cycles of 95°C for 30 s, 60°C for 1 min and 72°C for 30 s, on a Stratagene Mx3000P apparatus. RNA expression of the gene of interest was quantified in triplicate using the ΔCt method, and normalized to that of TBP and β-actin housekeeping genes using Qbase software.

### Cell proliferation, soft agar growth and wound healing assays

Cell proliferation rates were assessed by reduction of MTT (3-(4,5-dimethylthiazol-2-yl)-2,5-diphenyltetrazolium bromide) reagent [16]. Six-thousand cells were seeded in 12-well plates and cultured for 72 h. Viability was estimated at different time points. Briefly, 50 µl of MTT stock solution (5 mg/ml in PBS, pH 7.5) was added to each well at the indicated time points and incubated for 4 h at 37°C in the darkness. Then, 500 µl of solubilizing solution (0.04 M HCl in isopropanol) was added and incubated for 20 minutes at RT. Plates were read at 560 nm, and 640 nm for background subtraction, in a Beckman Coulter - Multimode microplate reader DTX-880.

For soft agar assay, a base layer of 1.5 ml of the corresponding culture media containing 0.5% agarose and 10% FBS was added to 35-mm plates. After the base layer was solidified, 5000 cells were resuspended in 1.5 ml of culture media containing 0.35% agarose and 10% FBS and added to the plates. Plates were incubated at 37°C in a humidified incubator for 14 d. Colonies were visualized and counted under florescence microcopy in an inverted microscope (Olympus, IX71).

For wound healing assay, cells were grown to confluence on 10 mm plates and wounded six times in the cell monolayer with a 200-µl standard pipette tip. Cells were then washed twice with PBS to remove cell debris and incubated with routine conditions. Images of the area of cell-free wounds were captured at 0, 2, 6 and 8 h. using an inverted microscope (Olympus, IX71) equipped with a digital camera (Olympus) under 100× magnification. To quantify the migration rate of the cells, the wound width was measured at ten different regions for each wound at each time point, and the mean and standard deviation were calculated.

### Apoptosis assay

Cells were seeded in triplicate on coverslips placed into 6-well plates and allowed to grow until the cell density reached 2.5×10^5^ cells per well (60% confluence). A 1 mM stock solution of staurosporin (STS) was prepared in DMSO, and added to the culture medium to give 1 µM STS final concentration. The cultures were subsequently incubated for different time periods. Terminal deoxynucleotidyl transferase-mediated dUTP (2′-deoxyuridine 5′-triphosphate)-digoxigenin nick end labeling (TUNEL) assay was performed on culture cells using the In Situ Cell Death Detection Kit (Roche), according to the manufacturer's instructions. Finally, coverslips were mounted on slides and stained with haemotoxylin. The percentage of apoptotic cells was calculated by determining the number of TUNEL positive cells in 10 randomly selected 60X fields using an optical microscope (Nikon, E100).

### GPAT2 overexpression

Human GPAT2 was stably overexpressed in MDA-MB-231 cells. To obtain stable cell lines, MDA-MB-231 cells were grown in 60-mm dishes to 90% confluence and then transfected with 5 µg of the cDNA encoding the complete open reading frame of human GPAT2 cloned in the pCMV6 vector (TrueORF, Origene) or with the empty vector as control. Both plasmids also contain a sequence coding for green fluorescent protein driven by an IRE translational element. Cells were transfected using Lipofectamine 2000 and then selected with Geneticin (Life Technologies) to establish pCMV6-GPAT2 (GPAT2-overexpressing) and pCMV6 (control) cells, which were used for MTT and TUNEL assays as described above.

Murine Gpat2 was transiently overexpressed in HeLa, Vero and HEK293 cells. Eight-thousand cells/well were seeded in 48MW plates and 24 h later cells were transfected with 0.6 µg/well of the cDNA encoding the complete ORF of murine Gpat2 cloned into pcDNA3.1 vector (pcDNA3.1-Gpat2) or the empty pcDNA3.1 vector as previously reported [4]. Forty-eight h later, cell density was assessed by crystal violet staining [16]. GPAT2 and Gpat2 overexpression was monitored by qPCR.

### Immunohistochemistry

To examine the expression of GPAT2 protein in human breast cancer tissue, we performed immunohistochemistry on a tissue microarray (TMA) of human breast cancers (n = 36) or normal tissue (n = 6) (Origene, CT565863). Endogenous peroxidase was inactivated by 1% H_2_O_2_ in methanol for 30 min. The slide was then washed three times with 1X PBS and blocked with 10% normal horse serum in 1% bovine serum albumin (Sigma) in 1X PBS for 1 h. The antigen retrieval was performed immersing the slide in 10 mM citrate buffer (pH 6) at 100°C for 5 min. After washing three times with PBS, the slide was incubated with rabbit anti-human GPAT2 (Sigma HPA036841) polyclonal antibody (1∶35) overnight at 4°C in a humidified chamber. Then, secondary HRP-conjugated anti-rabbit immunoglobulin diluted in the blocking solution (1∶150; Thermo-Pierce) was added for 1 h at room temperature. The reaction was developed with the LSAB2/HRP kit and liquid 3,3′-diaminobenzidine (Dako) according to the manufacturer's recommendations. Slides were counter-stained with haematoxylin to visualize the nuclei and analyzed with an Olympus BX52 microscope.

### 5-aza-2′-deoxycytidine (DAC) treatment

To determine the effect of demethylation on the expression of the GPAT2 gene, HEK293, HeLa, MCF7 and MDA-MB-231 cells were seeded at low density in six-well plates and treated with 2 µM DAC (Sigma) or DMSO for 96 h. After treatment, RNAs were isolated and analyzed for GPAT2 expression as described above.

### Athymic nude mice xenografts

Athymic female nude mice (N:NIH(S)-nu/nu/LP, 6 wk-old) were obtained from Facultad de Ciencias Veterinarias, UNLP (La Plata, Argentina). After a one-wk acclimation period, mice were randomly divided into three groups (shRNA-GPAT2, shRNA-Scr or control; n = 5 per group). Then, 3.2×10^6^ shRNA-GPAT2 or shRNA-Scr cells (suspended in 200 µl DMEM) were inoculated subcutaneously on the upper back of the mice; the control group received only the vehicle. Thereafter, mice were monitored daily for tumor occurrence by visual inspection and palpation. When detected, tumor growth was monitored twice weekly using calipers, and tumor volume was calculated using the following formula: length × width^2^ ×1/2. Twelve wk after cell administration, mice were euthanized and xenograft tumors were excised and weighed.

### Statistical analysis

Statistical comparisons were performed with SPSS statistics 17.0 software. The T-test or ANOVA, and the exact Fisher test were employed for continuous and discrete variables, respectively.

## Results

### GPAT2 is highly expressed in human testis and several cancer types

**Figure 1:**
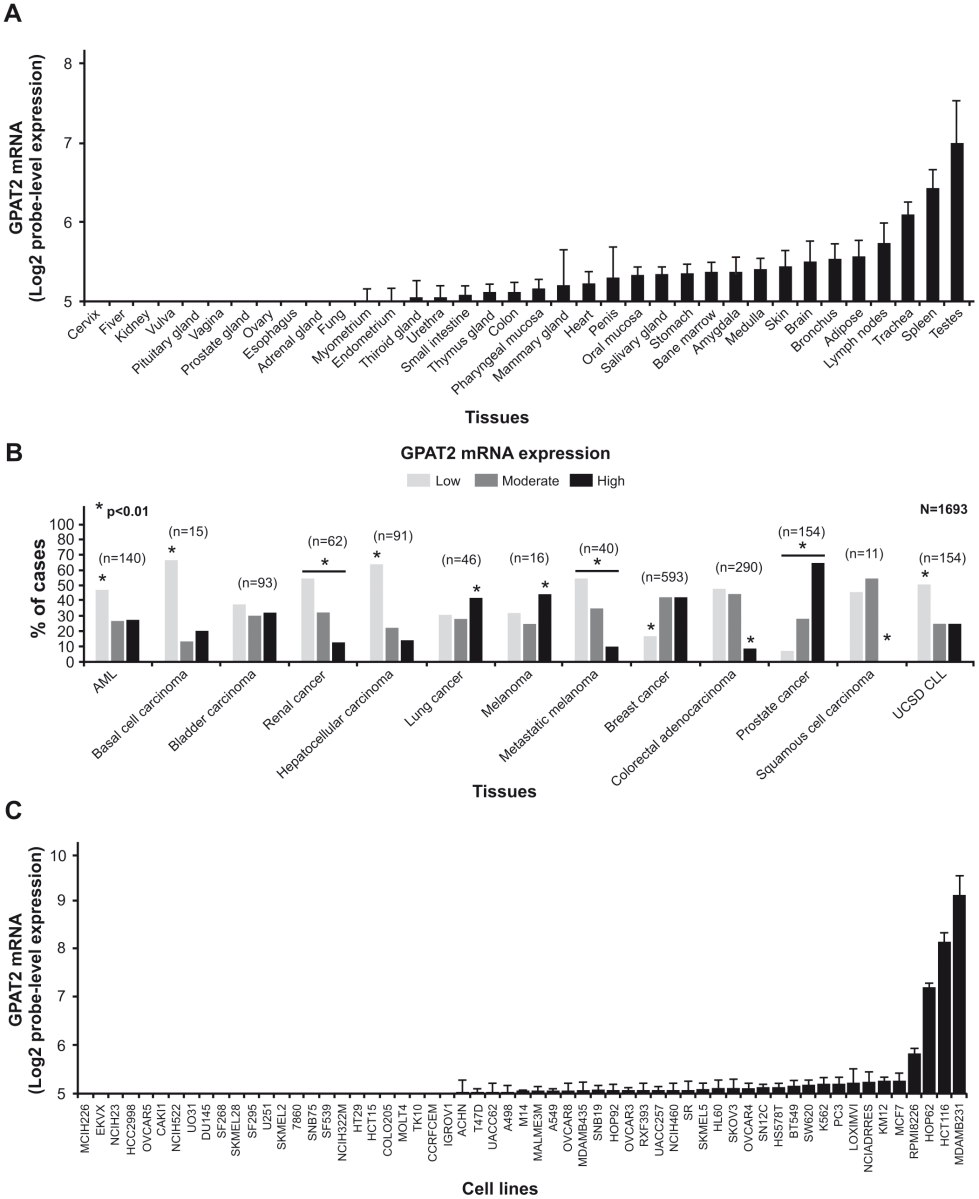
*In silico* analysis of GPAT2 mRNA expression profile. A) *In silico* analysis of GPAT2 expression profile across human normal tissues. B) GPAT2 mRNA expression across different tumor localizations was assessed with a bioinformatics approach, and expression level was classified into low, moderate and high. The percentage of cases in each category (low: light gray, moderate: dark gray, high: black) is displayed in the graph **p<0.01. C) GPAT2 expression profile across human cancer cell lines.

Because GPAT2 has been proposed to be a novel CT gene and in order to validate its high expression in human testis and in cancers, we performed an *in silico* analysis of GPAT2 mRNA expression. First, we evaluated the gene expression profile in 36 different normal human tissues and confirmed that the highest expression of GPAT2 was in testis (p<0.01, Figure 1A). The expression profile obtained from this analysis allowed us to classify GPAT2 expression as “testis-selective” [17].

To determine out which tumor locations express high GPAT2 mRNA levels, we analyzed a compiled dataset of 1693 samples derived from 13 different cancer types (Figure 1B). According to GPAT2 expression, samples were divided into three categories: low, moderate and high as detailed in the previous section. Statistical analysis revealed that tumor locations with higher percentage of samples in the “high GPAT2 expression group” (p<0.01) were: melanoma (44%), lung (41%), prostate (65%), and breast tumor (42%). On the other hand, renal (55%), colorectal (47%), hepatocellular (64%), basal cell (67%) and hematological cancers (AML, 47%; UCSD CLL, 51%) showed a significantly higher percentage of samples with low expression of GPAT2 (p<0.01). We also evaluated GPAT2 expression profile in cancer cell lines in order to get an *in vitro* model of GPAT2 expressing cell line. Fifty nine cell lines derived from 9 different cancers were analyzed. GPAT2 showed the highest expression level in the breast cancer cell line MDA-MB-231, which is characterized as a very aggressive tumor, because it is highly proliferative and tumorigenic (Figure 1C). This cell line was therefore chosen as our primary *in vitro* model to evaluate the phenotypic consequences of GPAT2 silencing.

### GPAT2 promotes cell proliferation, anchorage-independent growth, migration and survival of MDA-MB-231 cells

**Figure 2:**
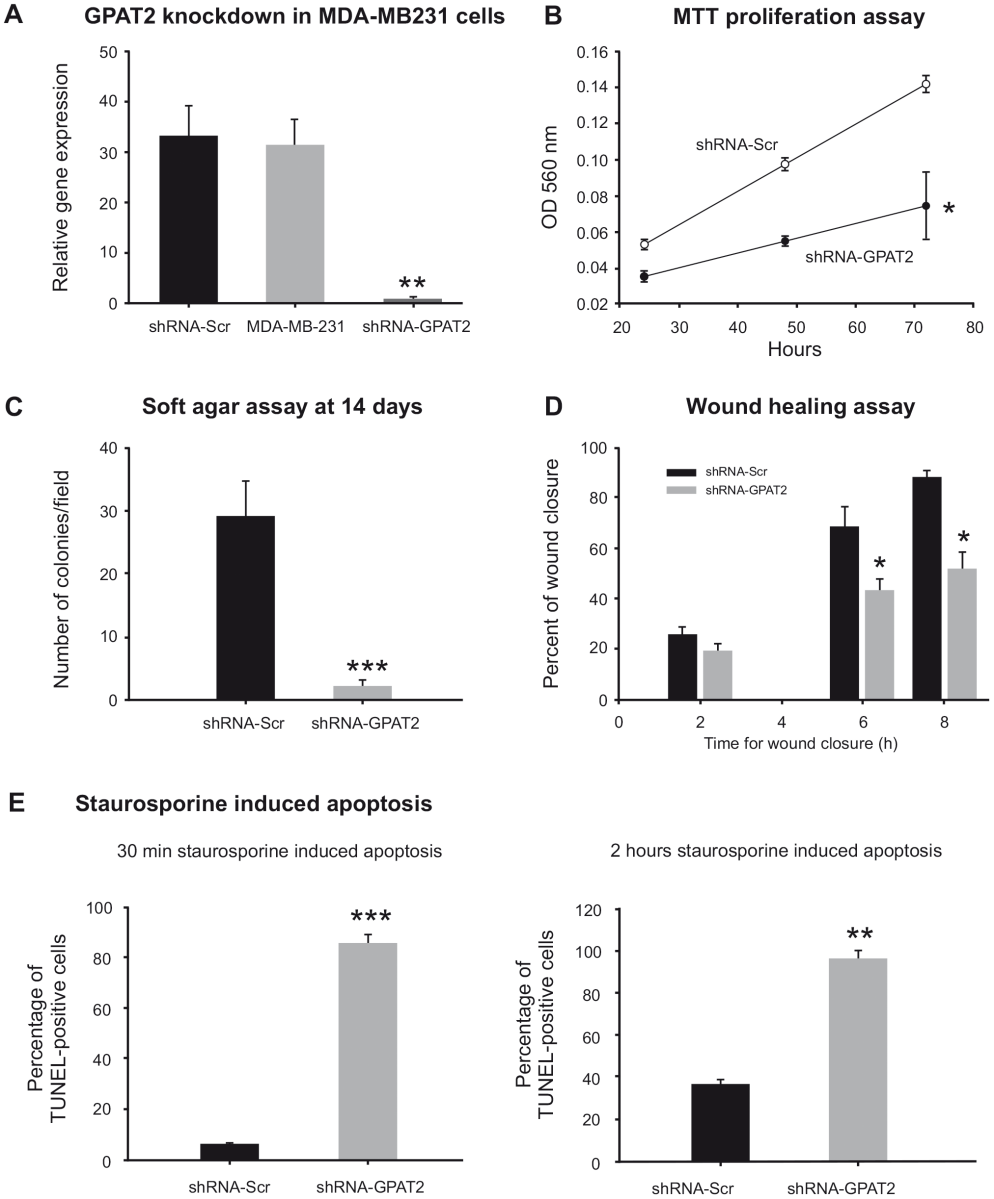
Phenotypic consequences of GPAT2 knock down in MDA-MB-231 cells. A) Total RNA was extracted from the MDA-MB-231 parent cell line, shRNA-Scr and shRNA-GPAT2 cells, subjected to cDNA synthesis and amplified by quantitative RT-PCR using primers for human GPAT2 gene, normalizing its expression level to that of TBP and β-actin housekeeping genes **p<0.01. B) shRNA-Scr and shRNA-GPAT2 cells were seeded at 10,000 cells/well on MW12 plates and incubated for 24, 48, and 72 h before estimating the cell proliferation rate by MTT proliferation assay *p<0.05. C) 5,000 cells from shRNA-Scr and shRNA-GPAT2 cells were seeded on 35-mm DMEM-agar plates and the number of colonies was quantified by fluorescent microscope after 14 d incubation under normal culture conditions ***p<0.001. D) shRNA-Scr and shRNA-GPAT2 cells were grown to confluence on 10 mm plates and the cell monolayer was wounded six times. The wound width was measured at 0, 2, 6 and 8 h under 100× magnification and the percentage of wound closure was calculated *p<0.05. E) shRNA-Scr and shRNA-GPAT2 cells were treated with apoptosis inducer staurosporine for 30 min or 2 h and the percentage of apoptotic cells was determined by counting the number of apoptotic and non-apoptotic cells using TUNEL assay and haematoxylin staining **p<0.01; ***p<0.001.

To analyze and compare different phenotypic consequences of GPAT2 stable silencing on MDA-MB-231, we used RNA interference technology to knock down GPAT2 expression and obtained two stable cell lines: MDA-MB-231 that expresses shRNA targeting GPAT2 (shRNA-GPAT2) and a control MDA-MB-231 cell line expressing a scrambled shRNA (shRNA-Scr). A 95% down-regulation of GPAT2 mRNA was obtained for shRNA-GPAT2, compared to shRNA-Scr cells (p<0.01, Figure 2A).

The MTT proliferation assay was used to test changes in cell growth rate. Interestingly, the proliferation rate of the GPAT2 silenced cells was 2-fold lower compared to the scrambled cells (p<0.01, Figure 2B). We also evaluated the anchorage-independent growth capacity, a main feature of malignant transformation, which measures the proliferation rate in a semisolid culture media. Silencing of GPAT2 markedly diminished the ability of MDA-MB-231cells to grow in a semisolid medium; the number of colonies counted at 14 days was reduced by 90% in shRNA-GPAT2 vs. shRNA-Scr cells (p<0.001, Figure 2C).

Cell migration was also diminished in the wound healing assay. The percentage of wound closure was lower in shRNA-GPAT2 vs shRNA-Scr cells at 6 h (43.3% vs. 68.7%; p<0.01), and 8 h (51.9% vs. 88.1%; p<0.01) after wound production (Figure 2D).

In order to learn whether GPAT2 silencing alters sensitivity to apoptosis, we treated shRNA-GPAT2 and shRNA-Scr cells with the apoptosis inducer STS (1 µM final concentration) for 30 min and 2 h. The percentage of apoptotic cells was determined by TUNEL assay (Figure 2E). At 30 min of treatment, apoptosis was dramatically increased in silenced cells compared to control cells (85.4% and 6.6%, respectively; p<0.001); this effect was also evident at 2 h with 96% and 37% mortality, respectively; p<0.01. The vehicle DMSO did not induce apoptosis in our assay conditions.

**Figure 3:**
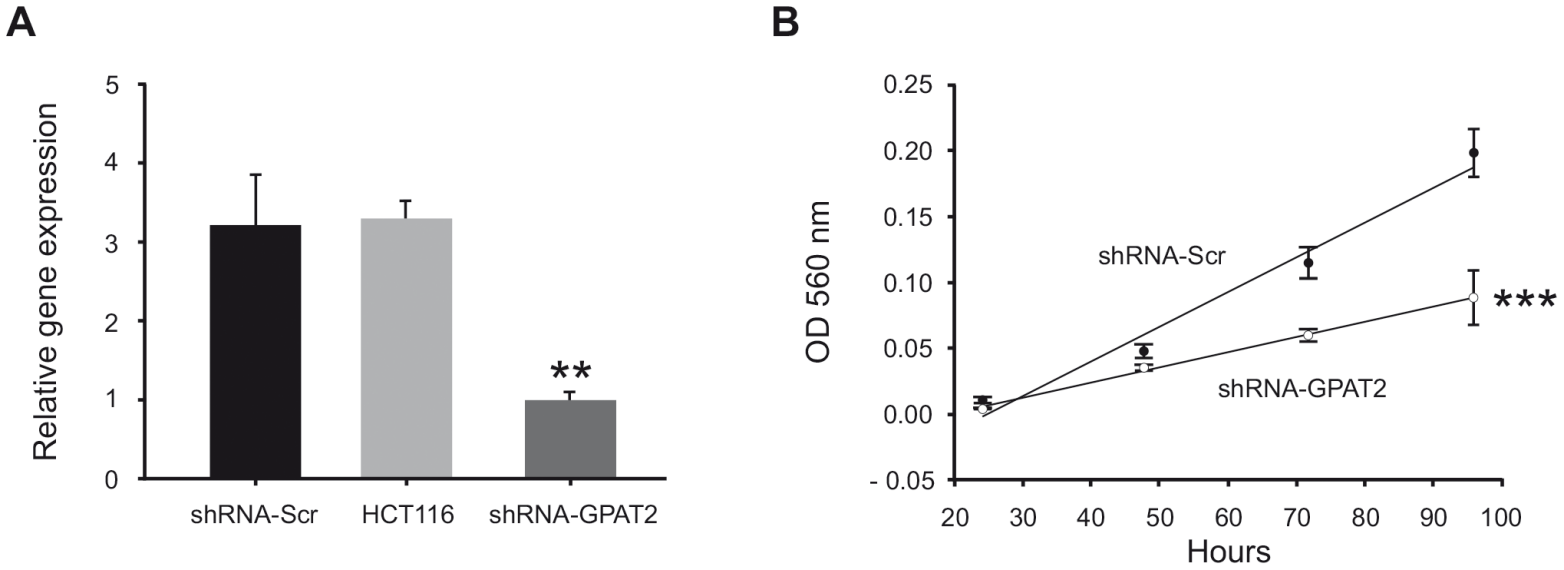
GPAT2 knock down in HCT116 cells. A) Total RNA was extracted from the HCT116 parent cell line, shRNA-Scr and shRNA-GPAT2 cells, subjected to cDNA synthesis and amplified by quantitative RT-PCR using primers for human GPAT2 gene, normalizing its expression level to that of TBP and β-actin housekeeping genes **p<0.01. B) shRNA-Scr and shRNA-GPAT2 cells were seeded at 5000 cells/well on MW12 plates and incubated for 24, 48, 72 and 96 h before estimating the cell proliferation rate by MTT proliferation assay ***p<0.001.

In order to check the phenotypic consequences of GPAT2 silencing in other cancerous cell line, HCT116 was chosen due to its high GPAT2 expression level (Figure 1C). GPAT2 mRNA was stably knocked-down by 70% (Figure 3A), and consistent with MDA-MB-231 cells, shRNA-GPAT2 cell line proliferation rate was lower than shRNA- Scr cell line (Figure 3B).

**Figure 4:**
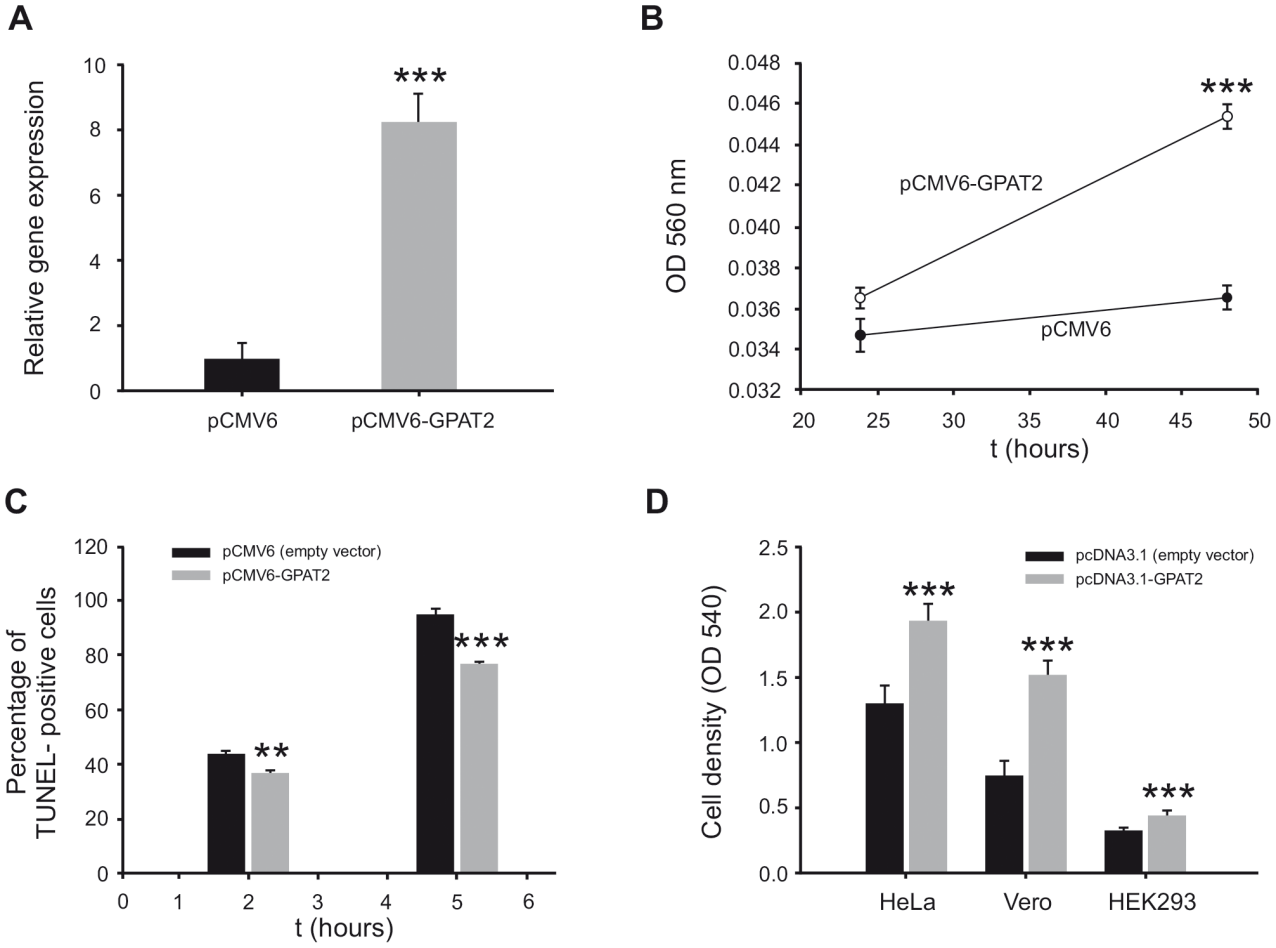
Phenotipic consequences of human and murine GPAT2 overexpression. A) Total RNA from pCMV6 and pCMV6-GPAT2 cells was extracted, subjected to cDNA synthesis and amplified by quantitative RT-PCR using primers for human GPAT2 gene, normalizing its expression level to that of TBP and β-actin housekeeping genes *** p<0.001. B) pCMV6 and pCMV6-GPAT2 cells were seeded at 10,000 cells/well on MW12 plates and incubated for 24, and 48 h before estimating the cell proliferation rate by MTT proliferation assay ***p<0.001. C) pCMV6 and pCMV6-GPAT2 cells were seeded in coverslips and 24 h later apoptosis was induced by 1 µM staurosporine treatment for 2 and 5 h. The percentage of apoptotic cells was determined by counting the number of apoptotic and non-apoptotic cells using TUNEL assay and haematoxylin staining **p<0.01; ***p<0.001. D) pcDNA3.1 (empty vector) and the cDNA coding for mouse Gpat2 cloned in pcDNA3.1 (pcDNA3.1-Gpat2) were transiently transfected in HeLa, Vero and HEK293 cells. Cell density was estimated 48 h post-transfection by crystal violet assay. ***p<0.001.

These results clearly showed that GPAT2 down regulation diminished cell proliferation and increased sensitivity to apoptosis of MDA-MB-231 cells. To test whether GPAT2 overexpression evokes a reverse phenotype we obtained a stable MDA-MB-231 cell line overexpressing GPAT2 (pCMV6-GPAT2) 8.5-fold higher than the empty-vector control. These cells proliferated 2-times faster than control cells (Figure 4A and B). STS-induced apoptosis was measured in pCMV6-GPAT2 and pCMV6 cells by TUNEL assay. When treated with 1 µM STS for 2 h, pCMV6-GPAT2 cells had a lower percentage of apoptotic cells than control cells (37% vs 44% p<0.01). After 5 h of incubation, 95% of control cells showed apoptotic traits whereas only 77% of GPAT2 overexpressing cells were affected, (p<0.001, Figure 4C), showing that pCMV6-GPAT2 cells were more resistant to STS-induced apoptosis. The effect of GPAT2 expression on cell proliferation was also evident when cDNA coding for murine Gpat2 was transiently transfected in different cell lines. Cell proliferation increased 68%, 100% and 48% when Gpat2 was overexpressed in HeLa, Vero and HEK293 cells respectively (Figure 4B).

### GPAT2 silencing inhibits MDA-MB-231 tumorigenicity *in vivo*

Results obtained *in vitro* led us to evaluate the tumorigenicity of the MDA-MB-231 shRNA-GPAT2 cells in nude mice. While 100% (5/5) of shRNA-Scr inoculated mice developed tumors, none of the mice given shRNA-GPAT2 (0/5) generated tumor xenografts. These results suggest that GPAT2 silencing severely inhibited the tumorigenicity of MDA-MB-231 cells.

### GPAT2 is highly expressed in undifferentiated breast carcinomas

**Figure 5:**
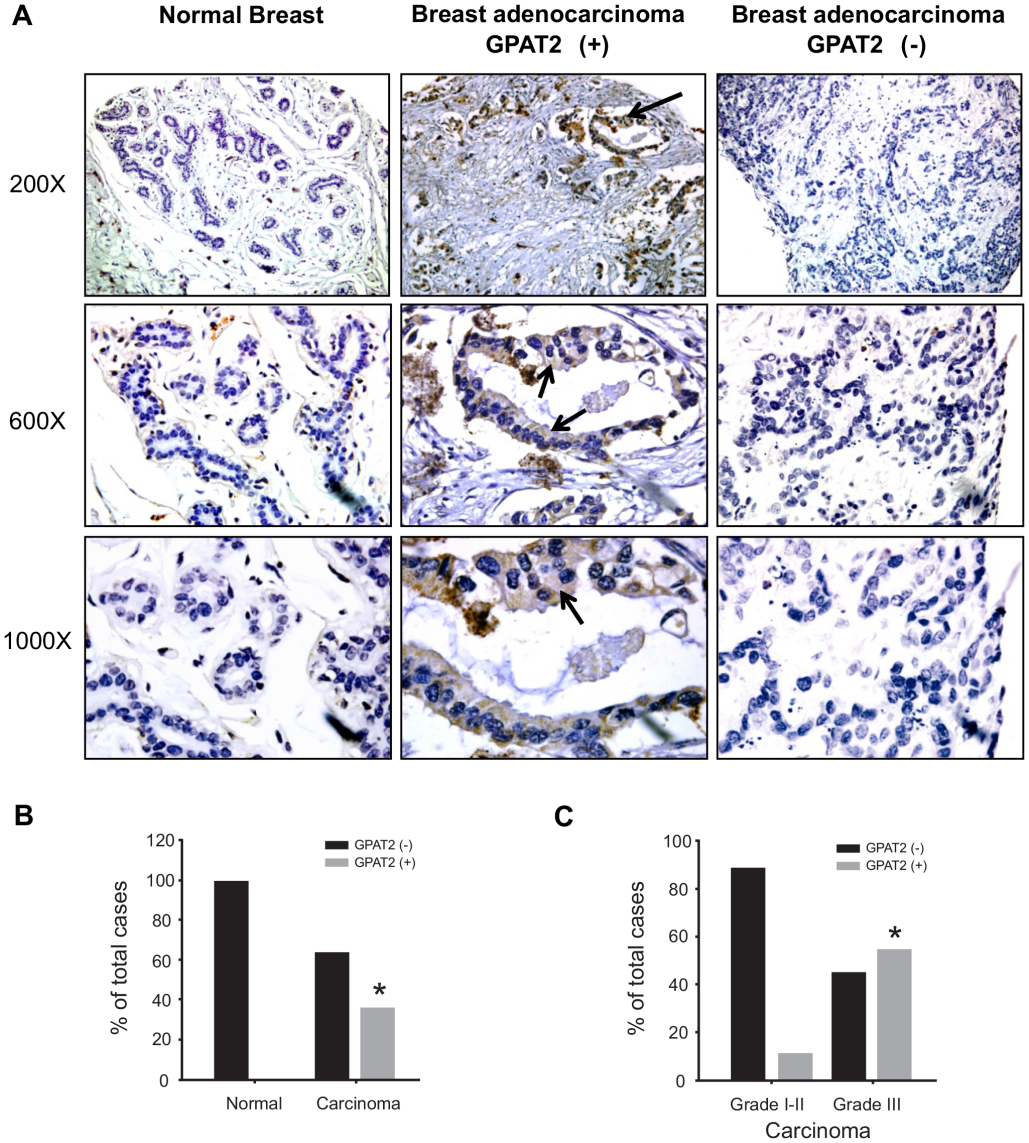
GPAT2 protein expression in human breast carcinomas. GPAT2 protein expression in human breast tissues was assayed on a tissue microarray (TMA) by immunohistochemistry. A) Representative samples of normal breast (left panel), breast adenocarcinoma positive for GPAT2 stainning (GPAT2 (+)) (middle panel) and breast adenocarcinoma negative for GPAT2 staining (GPAT2(-)) (right panel) are displayed. GPAT2 expression was detected by peroxidase reaction (brown signal, arrows) and nuclei were counterstained with haematoxilin (blue stain). Magnification: 200×, 600× and 1000×. Statistical analysis of GPAT2 protein expression on the TMA: B) frequency of GPAT2 expression between normal breast and breast adenocarcinoma (carcinoma) and C) frequency of GPAT2 expression in adenocarcinoma (carcinoma) samples according to their histological grade (Nottingham scale).

We showed that GPAT2 expression is required for the high rate of proliferation, anchorage independent growth, tumorigenicity, and survival of MDA-MB-231 cells. To analyze GPAT2 expression at the protein level in human breast adenocarcinomas, we performed immunohistochemistry (using an anti-GPAT2 antibody previously validated in our lab [4]) on a commercial breast tissue microarray. GPAT2 was not detected in any of the normal samples (n = 6), but its frequency in carcinomas (n = 35) was 37% (Figure 5A and B). Similar to its subcellular localization in normal testicular germ cells, GPAT2 showed immunoreactivity in the cytoplasm of cancerous cells (Figure 5A, middle panel). When the histopathological variables were analyzed, a significant positive association with the histological grade was obtained. Only 11% of Grade I/II tumor samples were positive for GPAT2 protein expression, compared to 55% for Grade III samples (p<0.05) (Figure 5C).

### GPAT2 expression is upregulated by DAC treatment

**Figure 6:**
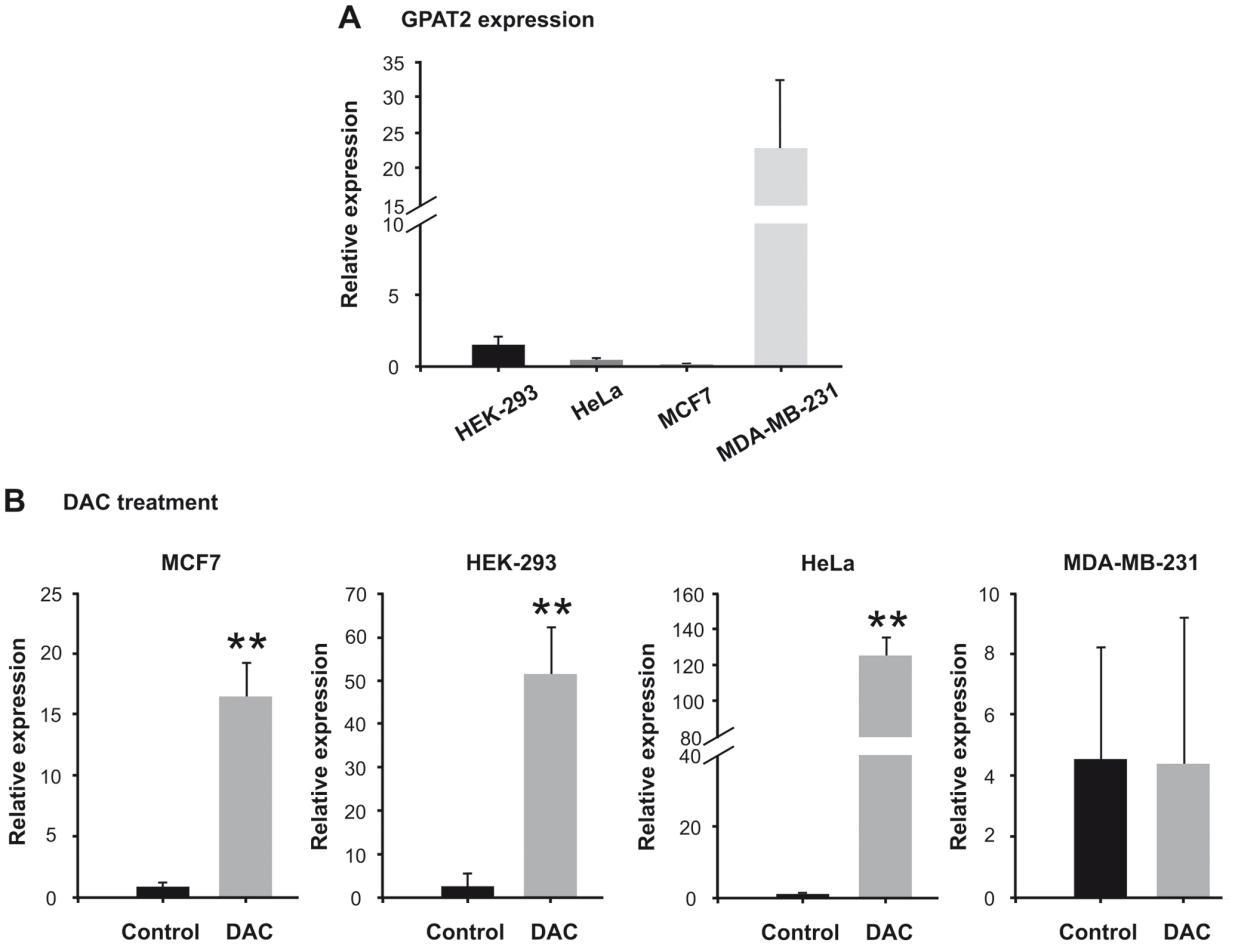
Effect of DAC treatment on mRNA GPAT2 expression in human cell lines. A) Relative mRNA expression of GPAT2 in human cell lines was assayed by quantitative RT-PCR. B) MCF7, HeLa, HEK-293 and MDA-MB-231 cells were treated with the methyltransferase inhibitor 5-aza-2′-deoxycitidyne 2 µM for 96 h (DAC) or with DMSO (control), and the mRNA relative expression of GPAT2 gene was assessed by quantitative RT-PCR. **p<0.01.

Epigenetic regulation is a hallmark of cancer testis gene expression [18]. To assess whether GPAT2 expression is regulated by DNA methylation, we treated HEK293, HeLa, MCF7 and MDA-MB-231 cell lines with the methyltransferase inhibitor DAC at 2 µM for 96 h or with DMSO as a control. The expression of GPAT2 was low in HEK293, HeLa and MCF7 cells compared to MDA-MB-231 cells (Figure 6A). DAC treatment was able to significantly increase GPAT2 expression in HEK293, HeLa and MCF7 cells, but had no effect on MDA-MB-231 cells (Figure 6B). This result strongly suggests that GPAT2 expression is epigenetically regulated.

### *In silico* co-expression analysis indicates that GPAT2 would be associated with piRNA metabolism and cell cycle control pathways

**Figure 7:**
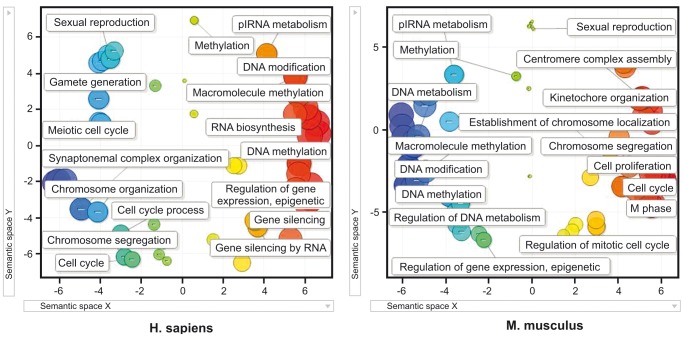
Gene ontology classification of genes co-expressed with GPAT2 in mouse and human testis. Scatterplot graph of the top 300 GPAT2 co-expressed genes showing the representative functional clusters according to gene ontology terms with a statistical significance of p<0.01, in a two dimensional space related to gene ontology terms' semantic similarities. Bubble color indicates the p-value of gene ontology terms (expressed as Log10 p-value), where blue and green bubbles are gene ontology terms with more significant p-values than the orange and red bubbles. Bubble size indicates the frequency of the gene ontology term in the underlying gene ontology database.7

Based on our experimental results and to identify the functional role of GPAT2, we performed a co-expression analysis in mouse and human testis. The top 300 best correlated genes (p<0.001) were selected for functional analysis. A visual summary of no redundant gene ontology terms was obtained by using DAVID and REVIGO bioinformatics tools. Remarkably, similar gene ontology terms were identified in mouse and human analyses (Figure 7). In both species, two clusters involving gene ontology terms connections were evident. One of these clusters, contained general terms like sexual reproduction and gamete generation, and included cell cycle-related gene ontology terms, like cell division, cell proliferation, cell cycle process, regulation of cell cycle and chromosome segregation. The other cluster was related to DNA metabolism including gene ontology terms like DNA methylation, regulation of gene expression, epigenetic, RNA biosynthesis, DNA modification and piRNA metabolism. Among the co-expressed genes with human GPAT2 included 18 CT genes, reinforcing the idea that GPAT2 behaves as CT gene.

## Discussion

The main finding of this study is that GPAT2 does not behave as a “classical” GPAT and its expression is linked to malignancy rather than to glycerolipid synthesis. We have previously reported in mice and rats that GPAT2 expression and activity is unrelated to the other three isoforms [4].

Human GPAT2 has been proposed as a novel CT gene candidate [6] based only on the fact that GPAT2 mRNA expression profile was selective to the testis and to myeloma cells. In order to validate this hypothesis, we have taken both *in silico* and experimental approaches to confirm that GPAT2 meets the criteria of a CT gene. We explored GPAT2 distribution in different cancer tissues, we detected GPAT2 expression at protein level in cancer cells and we obtained evidence that GPAT2 expression is epigenetically regulated.

One of the main features of CT genes is that they are expressed not only in testis but also in tumors. In this sense, tumors have been classified in high, moderate or low CT gene expressers [19]. Using data available in public databases, we analyzed the GPAT2 expression profile in human tumor samples and found that in each tumor location only a fraction showed high GPAT2 expression levels. The locations in which there was a statistically higher proportion of samples with “high expression” were lung, melanoma, breast, and prostate cancer, whereas “low expression” was most frequent in renal, colorectal, and hepatocellular cancers and in hematopoietic malignancies. This expression pattern in different tumors is consistent with the distribution of other CT genes in human cancers [19]. Although the number of CT genes identified has been increasing, knowledge of the expression pattern at the protein level is still limited. In this study, we explored GPAT2 protein expression in breast carcinoma tissues, and found that 36% of breast tumors express this protein. This frequency is relatively high compared to reported frequencies for other CT genes in breast cancer [19]. Additionally, when histopathological variables were analyzed, GPAT2 showed a positive association with the histological grade, which is also characteristic of CT genes, since they are preferentially expressed in high grade breast cancers [8], [20].

Another hallmark of CT genes is epigenetic regulation. CT genes have methylated promoters in normal non-expressing somatic tissues and are activated by demethylation during spermatogenesis and carcinogenesis [18], [20]. DAC treatment of human cell lines expressing GPAT2 at very low levels evoked a very strong induction of gene expression, whereas the same treatment had no effect on MDA-MB-231 cells, which normally express GPAT2. These results allowed us to conclude that methylation/demethylation is a mechanism governing GPAT2 expression.

One key question about CT genes is whether their expression contributes to the tumor phenotype. To address this, we stably knocked-down GPAT2 in MDA-MB-231 cells. Gene abrogation dramatically decreased cell proliferation, anchorage independent growth and migration of MDA-MB-231 cells, features that are all related to tumor progression. GPAT2 silencing also diminished cell proliferation of HCT116 cells and both murine and human GPAT2 overexpression increased cell proliferation. These results agree with experiments reporting either siRNA-mediated silencing or overexpression of specific CT genes, which demonstrate causality between CT gene expression and growth phenotype [21]–[25]. The dramatic reduction of the MDA-MB-231 tumor phenotype by GPAT2 silencing was also evident *in vivo*, because knocked down cells were unable to generate tumor xenografts in nude mice. The contribution of GPAT2 to malignancy appears to be not only restricted to an increased cell proliferation, because GPAT2 silenced cells were more sensitive to STS induced apoptosis and GPAT2 overexpressing cells were more resistant to STS induced apoptosis.

Our results, as well as those reported from other groups, support the idea that ectopic CT gene expression in somatic cells promotes tumorigenesis. The mechanisms by which each CT gene contributes to a tumor phenotype are poorly understood but revealing CT gene function in spermatogenesis could provide clearer insights into how CT genes act in cancer. For this reason, we performed a co-expression analysis for GPAT2 in mouse and human testis, the tissue in which GPAT2 exerts its physiological role. Two main clusters of ontological terms in both species were found. One of these clusters was associated with the regulation of gene expression by epigenetic mechanisms, RNA biosynthesis and piRNA metabolism, consistent with a report that GPAT2 plays a critical role in piRNA biogenesis [5]. piRNAs are small non-coding RNAs synthesized in germ cells, whose accepted function is to use DNA methylation to repress the expression in germ cells of deleterious retrotransposons [26]. The relationship between piRNA metabolism and cancer has been considered. In Drosophila ectopic expression of piRNA pathway genes are responsible for the development of brain tumors [27] and specific piRNAs are aberrantly overexpressed in gastric, colon, lung and breast cancer tissues [28]. However, the mechanisms of piRNAs involvement in tumorigenesis are yet to be determined. The second cluster showed ontological terms associated with cell cycle, such as M phase, chromosome segregation, centromere complex assembly and cell proliferation, suggesting a role for GPAT2 in the control of cell cycle, perhaps through its participation in chromosome segregation. Whether all these terms are associated with piRNA metabolism or if they reveal an independent function for GPAT2 must be determined, but a link between piRNA metabolism and mitosis was recently described [29].

In summary, our co-expression analysis in mouse and human testis confirm the role of GPAT2 in piRNA metabolism and suggest an emerging role for GPAT2 in the control of cell cycle, probably through its participation in chromosome segregation.

Our present data linking GPAT2 to tumorigenesis open the possibility of considering GPAT2 as a potential target for treatment of highly aggressive cancers.
